# On the Importance of Host MicroRNAs During Viral Infection

**DOI:** 10.3389/fgene.2018.00439

**Published:** 2018-10-02

**Authors:** Erika Girardi, Paula López, Sébastien Pfeffer

**Affiliations:** Architecture and Reactivity of RNA, Institut de Biologie Moléculaire et Cellulaire du CNRS, Université de Strasbourg, Strasbourg, France

**Keywords:** microRNA, virus, post-transcriptional regulation, defense mechanism, host–pathogen interaction

## Abstract

Every living organism has to constantly face threats from the environment and deal with a large number of pathogens against which it has to defend itself to survive. Among those, viruses represent a large class of obligatory intracellular parasites, which rely on their host machinery to multiply and propagate. As a result, viruses and their hosts have engaged in an ever-evolving arms race to be able to maintain their existence. The role played by micro (mi)RNAs in this ongoing battle has been extensively studied in the past 15 years and will be the subject of this review article. We will mainly focus on cellular miRNAs and their implication during viral infection in mammals. Thus, we will describe current techniques that can be used to identify miRNAs involved in the modulation of viral infection and to characterize their targets and mode of action. We will also present different reported examples of miRNA-mediated regulation of viruses, which can have a positive outcome either for the host or for the virus. In addition, the mode of action is also of a dual nature, depending on the target of the miRNA. Indeed, the regulatory small RNA can either directly guide an Argonaute protein on a viral transcript, or target a cellular mRNA involved in the host antiviral response. We will then see whether and how viruses respond to miRNA-mediated targeting. Finally, we will discuss how our knowledge of viral targeting by miRNA can be exploited for developing new antiviral therapeutic approaches.

## Introduction

Viral infections constitute a major threat for human health. As obligate intracellular parasites, viruses rely exclusively on the host cellular machinery to translate their genome, and therefore to replicate and propagate in their host and in the environment. *Per se*, they represent the ultimate example of selfish genes that are here solely to be amplified. Of course, this close dependency on a host organism also makes them vulnerable since they have to unveil their genome in the cells they infect. This results in an ongoing arms race in which the invading pathogen has to constantly evolve to find strategies to avoid detection and clearance by the host immune response. In parallel, the attacked organism has developed throughout evolution multiple ways to sense and fight back viral infections. As such, viruses can thus also be seen as crucial elements in the shaping of modern organisms.

In mammals, there are several layers of protection put in place to prevent the invading virus to establish a successful infection. One of the first line of defense is innate immunity, which is triggered by the recognition of foreign elements, brought in by the virus, and that include nucleic acids. In vertebrates, upon sensing of specific pathogen-associated molecular patterns, such as double-stranded (ds) RNA or the presence of a 5′ triphosphate group, the cell responds by the activation of a signaling cascade that results in the induction of type I interferon (IFN) expression, which in turn triggers transcription of hundreds of interferon-stimulated-genes (ISGs) that include pro-inflammatory cytokines (Nakhaei et al., 2009). This chain of events creates an antiviral state that interferes with viral replication, blocks protein synthesis, induces cellular RNA degradation and ultimately leads to apoptosis of the infected cell (Barber, 2001).

In other organisms, including plants, arthropods and nematodes, the presence of exogenous long double stranded (ds)RNA activates another mechanism, known as RNA interference (RNAi), which represents their major antiviral defense system. In this case, long viral dsRNA molecules are recognized and processed into small interfering (si) RNAs by the type III ribonuclease Dicer. These siRNAs are then loaded into effector complexes that invariably contain a member of the Argonaute family. The activated complex, termed RNA Induced Silencing Complex (RISC), can be directed via sequence complementarity toward target RNAs (in this case viral messenger or genomic RNAs) to mediate their cleavage and degradation (Ding and Voinnet, 2007). Whether such an RNA-based defense mechanism is still functional and relevant in vertebrates remains a debated topic that will not be addressed here. Nonetheless, it is now widely accepted that another class of small non-coding RNAs do play important roles during viral infections in mammals. Indeed, the same machinery that was originally designed to clear the cell of unwanted nucleic acids, is also involved in the biogenesis of miRNAs, which have emerged in the last 15 years as one of the more relevant species of regulatory RNAs in higher eukaryotes.

**FIGURE 1:**
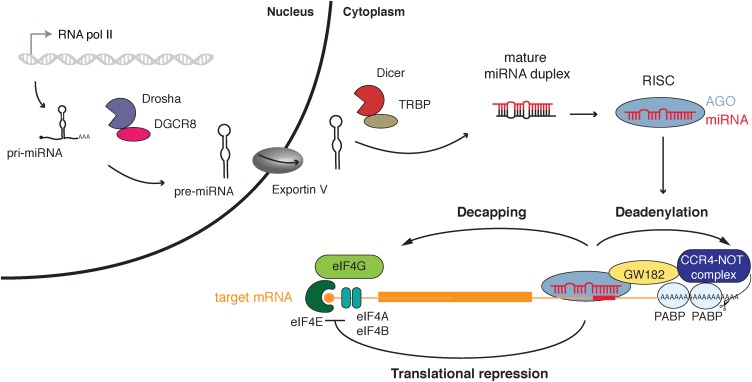
miRNA canonical biogenesis and function. miRNA genes are transcribed by the RNA polymerase II into a primary precursor (pri-miRNA) which is processed in the nucleus by the Microprocessor (Drosha and its cofactor DGCR8) to produce a hairpin structure precursor (pre-miRNA) that will be exported to the cytoplasm by Exportin 5. The pre-miRNA is processed in turn by Dicer into the mature miRNA duplex that will be loaded in an Argonaute protein (AGO) within the RNA induced silencing complex (RISC). One of the strands remains bound to Ago and the complex can mediate post-transcriptional gene regulation by targeting mRNAs through binding of the miRNA seed region (nucleotides 2–8) to the target mRNA (binding site represented by a red rectangle). Adaptor protein GW182 is recruited by RISC and can interact with polyA-binding proteins (PABP) inducing recruitment of CCR4-NOT deadenylase complex. The target mRNA is destabilized by deadenylation and decapping leading to its degradation. Translation of targeted mRNAs is also repressed by inhibition of the preinitiation complex assembly.

In the canonical biogenesis pathway, miRNAs are transcribed by RNA Polymerase II into a primary precursor, called pri-miRNA, processed by Drosha and its co-factor DGCR8 into a precursor of about 70 nucleotides (nt). The pre-miRNA is then exported by the Exportin 5 protein from the nucleus into the cytoplasm, where it will be cleaved by Dicer into a ∼22 nt long miRNA duplex. One of the two strands is selected and loaded into an Argonaute protein (in human AGO1 to 4), to form the basic RISC. The mature miRNA functions as a sequence-specific guide to trigger the effector complex onto the 3′ untranslated region (UTR) of the target messenger RNAs (mRNAs). Upon binding to its target RNA, the Argonaute protein can then bind an adaptor protein, known as GW182 or TNRC6, which in turn interacts with factors that act on mRNA translation and stability [see **Figure 1** and Bartel, 2018 for review].

It is generally accepted that the main determinant of miRNA sequence specificity is its seed region. Initially discovered purely based on bioinformatic evidence (Lai et al., 2005; Lewis et al., 2005) before the determination of AGO2 structure brought more evidence (Schirle and MacRae, 2012), the minimal seed corresponds to a short region at the 5′-end of miRNAs (nucleotides 2–7), which displays perfect complementarity with its target site (referred to as the seed-match). The 8mer sites with an additional match to nucleotide 8 and an A in position 1 are the most effective canonical sites and those identified with increased confidence by target-prediction tools (Bartel, 2009; Agarwal et al., 2015). In some cases, additional base pairing toward the 3′ end of the mature miRNA (the 3′-compensatory site) may compensate for suboptimal pairing in the seed region (Grimson et al., 2007; Broughton et al., 2016). The definition of “seed rules” was extremely important to enable target predictions. Given the limited size of the interaction sequence, the regulatory potential of miRNAs is extremely flexible. Indeed, conservative estimates indicate that at least 60% of the human coding genome might be regulated by miRNAs (Friedman et al., 2009). This also means that miRNA-mediated regulation can in theory be expanded to every source of exogenous target RNA. However, one should be careful as to not extrapolate that all these potential targets are physiologically meaningful as recent reports indicate that only a handful of them could indeed have a true impact (Rausch et al., 2015; Denzler et al., 2016; Pinzón et al., 2017).

In this review, we focus on the involvement of cellular miRNAs in viral infection in mammals. We describe the current methods for identification of proviral and antiviral miRNAs and their targets. We review the principal mechanism of action of miRNAs which modulate viral infection and we examine the means employed by viruses to subvert or induce miRNA effects. Finally, we discuss about the miRNA-based therapeutic strategies as a promising emerging field in the context of infectious diseases and viral vector therapy.

## Finding the Needle in a Haystack: Which miRNAs are Involved in Viral Infection

According to the latest release of miRBase^1^, the human genome contains almost 2000 miRNA precursors (Kozomara and Griffiths-Jones, 2014), each of which with the potential to regulate tens to hundreds of different targets, among them viral transcripts. Thus, finding the miRNAs playing important roles during a given virus infection can quickly become overwhelming. Conceptually, there are two main ways to identify these candidate miRNAs. One can first check if the infection has an impact on the miRNA profile of the cell or tissue studied. Indeed, if some miRNAs are strongly deregulated upon infection, it can be assumed that they might play a role during the viral cycle. The main limit with this approach though, is that it is impossible to know whether the observed miRNA regulation, which is a consequence of virus infection, is indeed meaningful for the virus, or if it is just an indirect effect without importance. The other possible approach is to go for a phenotypic screen in order to test, exhaustively if possible, the effect of overexpressing or blocking individually each miRNA on virus accumulation. This method has the advantage to be truly unbiased, but does have its limitation, especially concerning the blocking of miRNAs. Indeed, a specific cell line only expresses, at a functional level, a 100 different miRNAs at most, which means that it is easy to overlook the effect of inhibiting a miRNA that is not naturally expressed in the cells used for the screen.

### Impact of Viral Infection on miRNA Expression

**FIGURE 2:**
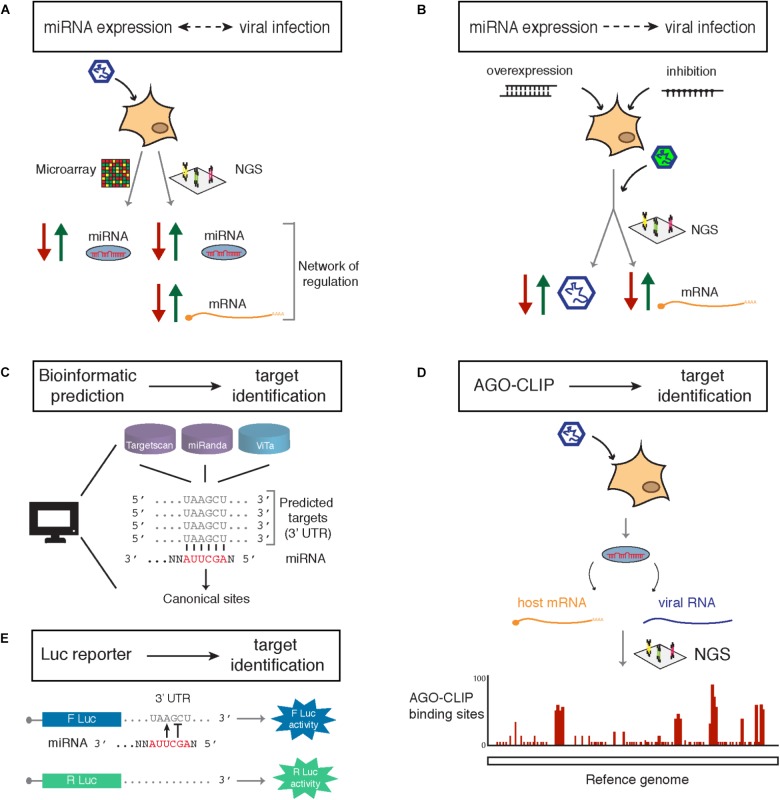
Current approaches available for identification of miRNAs involved in the regulation of viral infection **(A,B)** and their targets **(C–E)**. **(A)** Reciprocal regulation of miRNA expression and viral infection allows identification of candidate miRNAs upregulated (green arrow) or downregulated (red arrow) by miRNA profiling through microarray. Sequencing (next generation sequencing, NGS) provides further information on regulated targets and reveals networks of gene regulation. **(B)** Virus-centered phenotypic approaches are based on miRNA regulation of the infection. Screens based on the overexpression or inhibition of candidate miRNA in the context of infection, generally using a reporter virus (indicated by the green color), allow a direct observation of the effect on the viral accumulation. This approach coupled to transcriptome profiling also identifies target genes of candidate miRNA. **(C)** Computational analysis for target identification of a given miRNA are based on the identification of seed-matches in the 3′ UTRs of cellular mRNAs. Bioinformatic predictions rely on the use of target prediction tools such as Targetscan or miRanda for cellular targets or ViTa for viral genomes and transcripts. **(D)** Biochemical isolation of AGO crosslinked to the miRNA and bound target followed by deep sequencing (AGO-CLIP) allows identification of miRNA specific targets, either cellular or viral, in a genome-wide manner and reveals the precise binding sites on the target. **(E)** Luciferase (Luc) reporter assays allow functional validation of a miRNA binding site based on the measure of the luciferase enzymatic activity when a potential binding site is present on the 3′UTR. Variants of luciferase (F, firefly; or R, Renilla) containing or not the binding site are used to estimate differential regulation.

Although a number of changes induced by viral infection on miRNA expression is most likely indirect, in some cases monitoring these variations can prove very informative. There are different techniques available to measure the impact of viral infection on miRNA expression (**Figure 2A**). The throughput of the classical ones (northern blot analysis, RT-qPCR and *in situ* hybridization) is generally not sufficient to determine the complete miRNA profile of a sample. Therefore, one should rely on the use of multiplexed RT-qPCR, microarrays or Next Generation Sequencing (NGS)-based genome-wide approaches to get a complete picture of the level of expression of every miRNA.

Li et al. (2017) performed miRNA profiling in cells infected with HCV using NanoString nCounter miRNA expression assays and microarray analysis. Among the modulated miRNAs, the top hits miR-25, miR-130a/b, and let-7a were downregulated by the virus, both in cultured cells and liver tissues of infected patients, suggesting that HCV counteracts their proven antiviral capacity by reducing their levels (Li et al., 2017). Profiling of 250 miRNAs in enterovirus (EV)71-infected cells by quantitative real-time PCR showed that miR-141 was induced upon EV71 infection. This miRNA turned out to be proviral (Ho et al., 2011). To identify the miRNAs involved in regulating antiviral signaling pathways, Ingle et al. (2015) performed microarray-based miRNA profiling in human cells infected with Newcastle disease virus (NDV). miR-485-5p was one of the most upregulated ones not only upon NDV infection but also in cells infected with Influenza A virus (IAV) H5N1 or transfected with a synthetic dsRNA, polyI:C (Ingle et al., 2015). Similarly, Rosenberger et al. (2017) used microarray to profile the expression of miRNAs in the lungs of mice infected with IAV and found miR-144 among the most significantly upregulated ones. Ectopic overexpression of miR-144 increased infectious virion production in cells infected not only with influenza virus but also with the negative-sense single-stranded (ss) RNA vesicular stomatitis virus (VSV) and the positive-sense ssRNA encephalomyocarditis virus (EMCV). In parallel, the transcriptome profile of influenza-infected wild-type and miR-144 over-expressing cells was compared and allowed the identification of the transcriptional network regulated by miR-144 (Rosenberger et al., 2017). In another study, both global cellular miRNA and mRNA expression was profiled in Japanese encephalitis virus (JEV)-infected human microglial cells using an Affymetrix microarray platform and identified key pathways associated with the differentially expressed miRNAs and inversely correlated mRNAs during JEV infection (Kumari et al., 2016).

The use of small RNA cloning and sequencing has been employed in several recent studies to identify up- or down-regulated miRNA upon infection with different viruses (Motsch et al., 2012; Oussaief et al., 2015; Chen et al., 2016; Lodge et al., 2017). However, these studies did not necessarily confirm whether the regulated miRNAs were having pro- or anti-viral roles. When miRNA profiling is coupled with regular transcriptome analysis, this can provide useful information regarding the networks of regulated genes upon viral infection. Indeed, it is generally observed that the expression of a miRNA and of its predicted targets tends to be positively or negatively correlated, suggesting a frequent coordination between transcriptional or post-transcriptional regulation of a miRNA and its targets in gene networks (Tsang et al., 2007). As we will see below, many examples of positive and negative regulatory feedback loops between viral levels and miRNA expression have been identified, using NGS approaches or other techniques such as multiplexed RT-qPCR or microarray analyses.

### Virus-Centered Phenotypic Screens

Viruses engineered to express a reporter protein (e.g., GFP) have been used as functional reporters to follow pro- and anti-viral miRNA activity in gain- and loss-of-function studies (**Figure 2B**). In general, the reporter activity can be measured upon overexpression or inhibition of miRNA expression, thereby providing a proxy to directly assess the impact on virus accumulation (see **Table 1** for currently available overexpression and inhibition tools).

**Table 1:** Main tools to study miRNA gene function.

**miRNA overexpression**	miRNA mimic	Synthetic double-stranded RNA molecule mimicking the miRNA duplexes produced after Dicer processing. Designed to efficiently favor the loading of one miRNA strand (miR-5p or -3p) as a functional mature miRNA strand into RISC. Transient expression.
	Vector-based miRNA expression	miRNA precursor under the control of a strong RNA Pol II or Pol III promoter, processed by the biogenesis machinery. Expression of the miRNA often coupled with a fluorescent protein marker. Constructs can be cloned in lentiviral or adenoviral vectors to be packaged into viral particles to target hard-to-transfect cells or for *in vivo* purposes. Can be used for stable expression.
**miRNA inhibition**	AntimiR	Chemically modified, single-stranded antisense oligonucleotide that inhibits miRNA function by sequence complementarity. Modifications at the 2′ ribose position such as 2′-*O*-methyl and 2′ Fluoro (2′F)-RNA increase binding affinity and stabilize the molecule. A phosphorothioate backbone can be used to stabilize the molecule.
	Antagomir	2*’-O-*methyl modified antisense single-stranded RNA oligonucleotide, conjugated to cholesterol in 3′ end, that inhibits miRNA function by sequence complementarity. Developed as a pharmacological approach for silencing miRNAs *in vivo* (Krutzfeldt et al., 2005).
	*Locked nucleic acid (LNA)*	Chemically modified antisense RNA analog, in which the ribose sugar is locked by a methylene bridge joining the 2′-oxygen and 4′-carbon of the ribose to increase stability and specificity. The strong binding properties of LNAs make them particularly useful in anti-miRNA applications.
	*miRNA sponges*	Transcripts containing multiple tandem perfectly or imperfectly binding sites to a miRNA of interest. Act as competitive inhibitors of miRNA function. Can be engineered as fusions to a transgene in plasmid constructs via a strong promoter (Ebert and Sharp, 2010). Can be used either for transient or long-term loss-of-function studies both *in vitro* and *in vivo.*
	Decoy/tough decoy RNA	Antisense single-stranded RNA containing a microRNA binding domain (Decoy) or a stabilized stem-loop with two microRNA binding domains (TuD). Usually expressed from a strong Pol III promoter. Sequesters the miRNA into stable complexes through complementary base-pairing (Xie et al., 2012).
	Morpholino	Phosphorodiamidate morpholine oligomer (or morpholino) is an uncharged DNA analog in which morpholine rings replace the sugar moieties and non-ionic phosphorodiamidate linkages replace the phosphate linkages. Neutral charge of backbone reduces non-specific interactions with proteins.

Santhakumar et al. (2010) used either miRNA mimics or inhibitors and monitored viral growth by using viruses from all three herpesvirus families (α, β, γ) that encode green fluorescent protein (GFP) reporters. They identified host miRNAs with broad pro- and anti-viral properties, among which miR-199a-3p which leads to decreased viral growth in all three herpesviral subfamilies (Santhakumar et al., 2010). To test the extent of such miRNA antiviral activities in other viral infections, the same group conducted a screen on IAV and Respiratory Syncytial Virus (RSV) in human cells (McCaskill et al., 2017). Also, microscopy-based screen using miRNA mimics upon Dengue Virus (DENV), West Nile virus (WNV), and Zika Virus (ZIKV) identified several antiviral miRNAs against flaviviruses, including the miRNA miR-34, miR-15, and miR-517 families (Smith et al., 2017).

## Identification of miRNA Targets

Once a candidate miRNA has been found, it becomes essential to identify its targets to understand the molecular mechanisms underlying the effect on the virus. This can prove as difficult if not more as the initial identification of the miRNAs involved in regulating the virus of interest. Indeed, as mentioned above, miRNAs bind to their targets with limited complementarity, and the effect of their binding on the target RNA expression is usually mild. Several methods have been employed for miRNA target identification in vertebrates. The very same approaches are applicable to cellular targets involved in viral infection. However, they cannot all be directly transposed to the identification of virus-encoded targets, in case of a direct effect of the miRNA on a viral RNA.

### Bioinformatic Predictions

A large set of miRNA target prediction tools based on sequence conservation and seed complementarity to 3′ UTR of host coding genes has been developed, such as TargetScan (Lewis et al., 2003, 2005), miRanda (John et al., 2004), PicTar (Krek et al., 2005), DIANA-microT (Kiriakidou et al., 2004) (**Figure 2C**). Although computational approaches are undoubtedly valuable in preliminary identification of miRNA target genes, they do not always identify the actual interaction between a miRNA and its target. Moreover, predictions do not include information about expression levels of miRNAs or their targets, leaving the question open about whether the miRNA-mRNA interaction is biologically relevant. Finally, the existence of non-canonical miRNA-target interactions extends the number of potential targets which are not necessarily considered by all bioinformatic tools.

In addition to the aforementioned limitations encountered to find cellular targets, bioinformatics predictions become especially difficult for the identification of miRNAs bound directly to virus. The main reason is that most of the prediction algorithms rely on the cross-species conservation of miRNA binding sites to select for the ones that were maintained during evolution. Therefore, the viral genomes are not included in the commonly used target repositories. In addition, the miRNA target sites are not necessarily contained in the 3′UTRs as for endogenous host targets. However, one group developed ViTa, a database for cellular miRNA targeting virus genomes and virus transcripts. This database contains information about known host miRNAs, known viral miRNAs, known and putative host miRNA target sites on viruses. It provides information such as human miRNA expression, virus tropism and virus comparisons (Hsu et al., 2007). Besides this report, initial efforts in the field identified first a biological effect by using gene reporter assays and then moved backward to the *in silico* search for identification of miRNA binding sites. Thus, in an early study, Dicer1-deficient mice were shown to be hypersusceptible to VSV infection, independently on RNAi or the interferon response. Fusing different portions of the VSV sequence in both positive (+) and negative (-) orientations to luciferase reporters, the authors identified the minimal region for regulation. Target prediction algorithms then allowed them to reveal potential target sites for miR-24 and miR-93 (Otsuka et al., 2007). Another example of prediction and identification of miRNA-viral target interaction concerns the antiviral miR-142 on Eastern equine encephalitis virus (EEEV). Target prediction algorithms identified binding sites for the haematopoietic-specific miR-142-3p in the 3′ Non-Translated Region (NTR) of the virus (Trobaugh et al., 2014). Indeed, EEEV is defective for replication in human and murine myeloid cells which express miR-142.

### Experimental Approaches to miRNA Target Identification

To increase the low signal-to-noise ratio inherent to purely computational prediction approaches, different attempts have been made to measure the global effect of altering the level of one candidate miRNA. Methods like microarray or high-throughput sequencing indeed provide indirect relationships between miRNAs and their targets. Experimental tinkering with endogenous miRNA expression should correspond to predictable changes in target expression, either at the RNA (Lim et al., 2005) or protein (Selbach et al., 2008) level. Thus, in order to identify cellular targets of miR-197 responsible for its antiviral effect on EV71, Tang et al. (2016) used a proteomics-based approach. McCaskill et al. (2017) and colleagues made use of a reverse-phase protein array (RPPA) to screen the expression levels of global signaling pathway markers to gain insights on the host pathways targeted by the miRNAs with antiviral properties against IAV. The advantage of conducting the RPPA is that it enables not only protein levels to be examined but also to distinguish the phosphorylation state relevant for the activation status of signaling pathways (McCaskill et al., 2017).

Other techniques provide a more solid evidence of what can be the target RNAs bound by miRNAs. These rely on the biochemical isolation of miRNA-target RNA complexes and can be either focused on one single miRNA (Easow et al., 2007), or more broadly on an Argonaute protein. The latter approach has been refined extensively in the past few years and is now based on the chemical cross-linking of one Argonaute protein to its target RNAs prior to its immunoprecipitation followed by deep sequencing (**Figure 2D**). There are several variations of this CLIP (Cross-Linking and ImmunoPrecipitation) technique, which allow the identification of miRNA target networks at a genome-wide level (Chi et al., 2009; Hafner et al., 2010). In addition to isolate physical interactions between the miRNA and its targets, these approaches provide deeper insights on the nature of binding site and in some cases can identify binding site locations with very high accuracy. CLIP data also revealed that a large portion of miRNA-target interactions *in vivo* are mediated not only through the canonical seed-match sites but also via non-canonical sites previously neglected by bioinformatic predictions. Interestingly, AGO-CLIP data are currently exploited to implement prediction tools. For instance, Agarwal et al. (2015) generated an improved quantitative model of canonical targeting based on available CLIP data. They showed that the vast majority of functional sites are canonical since non-canonical sites do not mediate detectable repression despite binding the miRNA. Of note, the appearance of databases such as miRTarbase which contains a curated collection of miRNA-target interactions with experimental support (Hsu et al., 2011; Chou et al., 2016) allows a step forward into the target validation compared to the mere prediction.

High-throughput sequencing of RNA isolated by crosslinking immunoprecipitation (HITS-CLIP) experiments of human AGO during HCV infection showed robust AGO binding to the HCV 5′UTR at known miR-122 sites. Moreover, Luna et al. (2015) describe that HCV serves as a sponge for miR-122 from endogenous targets, suggesting that bi-targeting of either cellular or viral RNAs is crucial for the final miRNA effect. A more recent paper characterized AGO binding landscapes for a number of medically important viruses (Scheel et al., 2016). Scheel et al. (2016) took advantage of an improved method of target identification based on miRNA-target chimera isolation in AGO, so called CLEAR-CLIP method (Moore et al., 2015) and showed a broad AGO coating for several RNA viruses.

Independently of the approach that is used to generate a list of putative targets, these have then to be experimentally validated. Reporter assays were among the first and more straightforward approaches to measure miRNA targeting (Doench and Sharp, 2004). The rationale behind the design of a miRNA reporter is very simple: the predicted miRNA target sequence is fused to the 3′UTR of a reporter gene and the reporter expression will be measured compared to a normalizer gene (**Figure 2E**). For a luciferase assay, the functionality of the target site can be monitored by measuring the luciferase enzymatic activity, while the expression of a normalizer gene will stay unaffected. Moreover, fluorescent reporters have also been chosen as functional read-outs of miRNA activity in living cells, working as biosensors for microscopy-based approaches and single-cell based analyses (Bassett et al., 2014). After the validity of the reporter regulation has been established, the effect of the miRNA on the endogenous target (either at the mRNA or at the protein level) should be measured, before ultimately assessing the effect of this target regulation on virus accumulation *in vitro* and/or *in vivo*.

## Examples of miRNA-Mediated Regulation of Viral Infection

**FIGURE 3:**
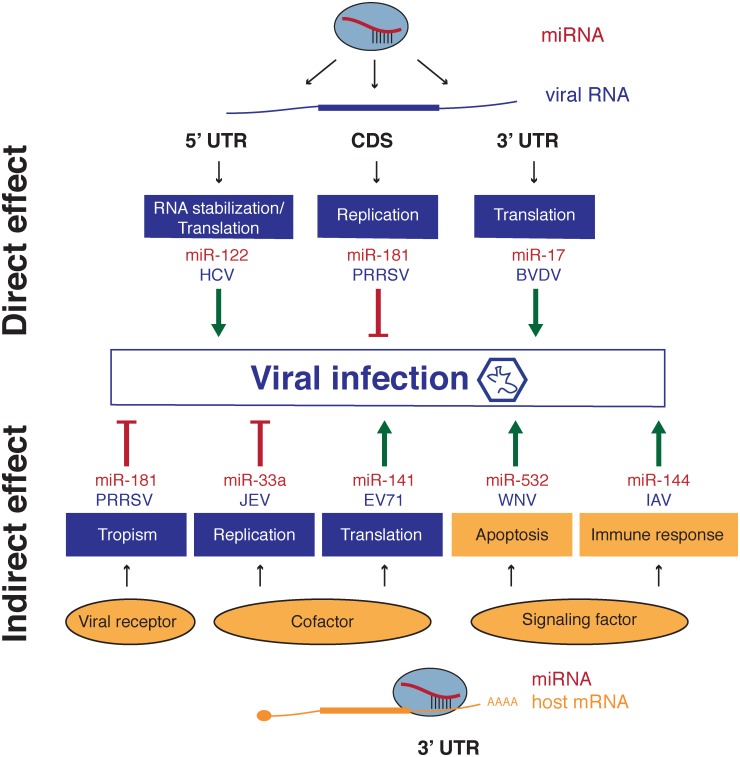
Mechanism of action of miRNAs which modulate viral infection. **(A)** miRNA direct effect on virus regulation takes place by direct targeting of viral RNAs at different regions such as 3′UTR, 5′UTR or coding sequences. Binding leads to RNA stabilization, enhanced translation or impaired replication. **(B)** Indirect effect involves modulation of expression of a cellular transcript encoding a host factor required for one or several steps in the viral cycle. Modulation of receptor expression regulates entry of the virus affecting tropism and cofactors required for replication complexes or translation can impair or enhance viral replication and viral protein production respectively. miRNAs also participate to enhance or restrain cell responses to the infection for instance immune response or defense mechanisms such as apoptosis induction. Viral cycle steps are represented in blue, while host factors and associated pathways are labeled in orange.

We have now seen multiple ways by which the identity and implication of specific miRNAs in the replication cycle of viruses can be unveiled and we have briefly mentioned a few of them. In this part, we will describe in more details some selected cases of both positive and negative regulation of viruses mediated by miRNAs. Some of these cases are described in **Figure 3** and a more complete list (although not exhaustive) can be found in **Table 2**.

**Table 2:** Examples of miRNA involved in viral infections.

microRNA	Virus	Effect	Target	Reference
miR-122	HCV	Proviral	Direct: viral 5′ UTR	Jopling et al., 2005; Lanford et al., 2010
miR-485	NDV and H5N1	Proviral/Antiviral	Indirect: RIG-I-mRNA Direct: H5N1 PB1 RNA	Ingle et al., 2015
miR-141	EV71	Proviral	Indirect: eIF4E mRNA	Ho et al., 2011
miR-142-3p	EEEV	Proviral/ Antiviral	Direct: viral 3′ NTR	Trobaugh et al., 2014
miR-17, let-7	Pestiviruses (BVDV)	Proviral	Direct: viral 3′ UTR	Scheel et al., 2016
miR-301a	JEV	Proviral	Indirect: IFN response	Hazra et al., 2017
miR-144	IAV, EMCV, VSV	Proviral	Indirect: TRAF6 mRNA	Rosenberger et al., 2017
miR-146a	HeV	Proviral	Indirect: RNF11 mRNA	Stewart et al., 2013
miR-24, miR-93	VSV	Antiviral	Direct: viral genes L and P	Otsuka et al., 2007
miR-221, miR-222	HIV-1	Antiviral	Indirect: CD4 mRNA	Lodge et al., 2017
miR-181	PRRSV	Antiviral	Indirect: CD163 mRNA	Gao et al., 2013
miR-181	PRRSV	Antiviral	Direct: viral ORF4	Guo et al., 2013
miR-130	PRRSV	Antiviral	Direct: viral 5′ UTR	Li L. et al., 2015
miR-542-5p, miR-24	IAV, RSV	Antiviral	Indirect: p38 MAPK pathway	McCaskill et al., 2017
miR-223	DENV-2	Antiviral	Indirect: STMN1 mRNA	Wu et al., 2014
miR-199, miR-214 and others	MCMV, HCMV, MHV-68, SFV	Antiviral	Indirect: ERK/MAPK, oxidative stress, and PI3K/AKT signaling	Santhakumar et al., 2010
miR-33a	JEV	Antiviral	Indirect: EEF1A1 mRNA	Chen et al., 2016
miR-34, miR-15 and miR-517	DENV, WNV, JEV	Antiviral	Indirect: Wnt pathway	Smith et al., 2017
miR-3614-5p	DENV	Antiviral	Indirect: ADAR1 mRNA	Diosa-Toro et al., 2017
miR-127-3p, miR-486-5p and others	IAV	Antiviral	Direct: viral genome	Peng et al., 2018
miR-25, Let-7, miR-130	HCV	Antiviral	Indirect: HCV co-factors	Li et al., 2017
miR-323, miR-491, and miR-654	IAV	Antiviral	Direct: PB1 RNA	Song et al., 2010
miR-532	WNV	Antiviral	Indirect: SESTD1 mRNA	Slonchak et al., 2016
Hs-154	WNV	Antiviral	Indirect: CTFC and ECOP mRNAs	Smith et al., 2012
miR-555	Poliovirus	Antiviral	Indirect: hnRNPC mRNA	Shim et al., 2016
miR-155	VSV, SeV	Antiviral	Indirect: SOCS mRNA	Wang et al., 2010
miR-197	EV71	Antiviral	Indirect: RAN mRNA	Tang et al., 2016

*HCV, Hepatitis C virus; NDV, Newcastle disease virus; H5N1, Influenza A virus subtype H5N1; EV71, Enterovirus 71; EEEV, Eastern equine encephalitis virus; BVDV, Bovine viral diarrhea virus; JEV, Japanese encephalitis virus; IAV, Influenza A virus; EMCV, encephalomyocarditis virus; VSV, Vesicular stomatitis virus; HeV, Hendra virus; HIV-1, Human Immunodeficiency Virus 1; PRRSV, Porcine reproductive and respiratory syndrome virus; RSV, Respiratory Syncytial Virus; DENV, Dengue virus; MCMV, Mouse Cytomegalovirus; HCMV, Human Cytomegalovirus; MHV-68, murine gammaherpesvirus-68; SFV, Semliki forest virus; WNV, West Nile virus; SeV, Sendai virus.*

### Direct Targeting of Viral RNA

The number of examples of viral regulation by direct binding of miRNAs targeting the viral genome remains limited. One possible explanation could be that if a miRNA target site with deleterious consequence for the virus would appear within a viral genome, the selection pressure would most likely remove this sequence quite rapidly in the virus progeny. This theory is backed up by the finding that a majority of direct host miRNA/viral RNA interactions results in a positive regulation of the viral cycle. In this case indeed, if the binding of the miRNA within the genome provides an evolutionary advantage and/or increases the viral fitness, then it will be maintained by the virus. However, there are some described cases where direct binding of a miRNA on a viral RNA does have a negative impact on the virus. As we will see, this can be explained by the mutual exclusion of the virus and the miRNA due to tightly controlled tissue-specificity. Actually, the idea that the tissue-tropism of some viruses can be partly due to miRNA expression is quite widespread.

The first discovered miRNA-virus interaction is the one involving miR-122 and HCV. HCV is a hepatotropic virus with a positive sense ssRNA genome. The liver-specific miR-122 is essential for the viral replication and positively regulates the virus by the direct interaction of the miRNA to the viral genome, which contains three different binding sites for miR-122 in the 3′ and 5′ UTRs. The regulatory function of miR-122 is exerted after binding to the 5′ UTR of the genome, upstream of the Internal Ribosomal Entry Site (IRES), and leads to increased viral RNA accumulation (Jopling et al., 2005) and enhanced viral protein translation after recruitment of the 48S ribosomal subunit (Henke et al., 2008). However, it seems that the dominant mechanisms leading to the positive effect of miR-122 binding is by protection of the genomic RNA 5′ extremity. Indeed, binding of miR-122-loaded AGO2 stabilizes HCV RNA by preventing its decay (Machlin et al., 2011; Shimakami et al., 2012) most likely caused by the XRN1 exonuclease (Li Y. et al., 2015). The positive effect of miR-122 on the virus certainly reflects the close co-evolution of HCV with its host and might be involved in defining the tropism of the virus, although it could also have appeared because of the liver tropism.

As mentioned previously, Scheel et al. (2016) performed a wide analysis of miRNA binding sites in several viruses using AGO2 CLIP. Among the tested viruses, they found that there were binding sites for the cellular miRNAs miR-17 and Let-7 in the 3′UTR of the bovine viral diarrhea virus (BVDV) genome (Scheel et al., 2016). Although the exact mechanism was not elucidated, the authors showed that this targeting of the BVDV genome had a positive impact on viral RNA and protein production. In addition, and similarly to HCV and miR-122, the sequestration of miR-17 by the viral RNA resulted in the de-repression of the miRNA cellular targets. These findings indicate that direct binding of viral RNAs by cellular miRNAs is more common than anticipated, although the physiological importance remains to be determined for a lot of these interactions.

Another example of a miRNA that partly plays a role in cell tropism is the haematopoietic lineage-specific miR-142, which restricts EEEV replication in myeloid cells. This miRNA binds directly to several conserved binding sites in the 3′UTR of the viral genome and thus restricts translation of non-structural proteins affecting subsequent viral replication in this lineage. Thus, the downregulation of virus accumulation in myeloid cells suppresses IFNα and β production, which allows the infection to occur and leads to the neuropathological features that characterize the viral disease (Trobaugh et al., 2014). Similarly, porcine reproductive and respiratory syndrome virus (PRRSV) can be directly targeted by both miR-181 and miR-130b cellular miRNAs. In this case as well these miRNAs can inhibit PRRSV replication. Studies on miR-181 family have shown that the target site is found on the region downstream of the ORF4 and inhibits the virus by targeting subgenomic RNAs. Expression of miR-181 family members is low in cells that are permissive for the virus. Over-expression of all four miRNAs belonging to this family inhibits PRRSV in a seed-dependent manner leading to 80% reduction in viral RNA accumulation and 30-fold decreased titers in porcine alveolar macrophages (Guo et al., 2013). Despite the efficient antiviral effect of different miRNAs on PRSSV, further investigation is needed to have a better insight of the mechanisms of action of this type of regulation.

In addition, cellular miRNAs can also be involved in regulating the switch from lytic to latent infection in herpesvirus-infected cells by targeting viral mRNAs. Thus, the neuronal specific miR-138 binds to and regulate the immediate early transcript ICP0 in herpes simplex virus 1 infected cells, thereby helping in maintaining latency (Pan et al., 2014). In a similar manner, miRNAs from the miR-200 family regulate latency of the human cytomegalovirus by targeting the viral UL122 transcript (O’Connor et al., 2014).

### Indirect Effect by Regulation of Cellular mRNAs

This form of viral regulation by host miRNAs is the most described in the literature. The indirect effect on the virus is in this case due to the targeting of mRNAs encoding host factors involved in one or several steps of the viral cycle or important elements in the establishment of the immune response and defense mechanisms.

#### Cellular Tropism and Viral Entry

The first step for a virion to start the infection is the permissiveness of a given cell by expression of viral receptors for entry. By regulating the expression of a receptor, miRNAs can therefore regulate the tropism of a virus for a cell type and influence the successful establishment of infection. The aforementioned virus, PRRSV, usually infects macrophages and dendritic cells and, to a lesser extent, monocytes. This tropism is in part due to the lower expression of the PRRSV receptor, CD163, at the surface of these latter cells, whereas it is expressed at higher levels once they differentiate into macrophages or dendritic cells. Gao and collaborators determined that miR-181 targets the 3′ UTR of CD163 mRNA and they showed an inverse correlation between the miRNA and the receptor expression levels. Through regulation of surface receptor expression, this miRNA hence affects the viral tropism and negatively regulates PRRSV (Gao et al., 2013). It is interesting to note that miR-181 is therefore exerting a dual action on this virus, both direct on the viral genome and indirect by regulation of CD163.

The Human Immunodeficiency Virus 1 (HIV-1) receptor, the CD4 surface protein, was also shown to be regulated by two miRNAs, miR-221 and miR-222. Interestingly, in this study, the authors found that the expression of these two miRNAs was low in productively infected macrophages, whereas it was higher in bystander macrophages. They also linked their upregulation to TNFα production in these cells, and confirmed that miR-221 and miR-222 could restrict entry of HIV-1 by downregulating CD4 expression (Lodge et al., 2017). Furthermore, the HIV-1 tropism is also determined by two different co-receptors, CCR5 or CXCR4. A signaling cascade involving miR-146a regulates CXCR4 expression in resting CD4+ T lymphocytes, making them less susceptible to HIV-1 infection. However, after T cell activation, the transcription factor PLZF downregulates the expression of miR-146a expression, leading to the expression of CXCR4 and hence rendering the cells susceptible to infection (Quaranta et al., 2015).

#### Cellular Cofactors Involved in Viral Replication

Another level where the viral cycle can be successfully impaired is at the replication step by regulation of cellular cofactors essential for the production of new copies of viral genomes. This is the case during infection by the flavivirus JEV. Chen et al. (2016) found that the expression of miR-33a was repressed in cells infected with JEV, and that one of the targets of this miRNA was the elongation factor EEF1A1. Interestingly, the authors went on to show that EEF1A1 interacts with components of the replication machinery and contributes to the stabilization of the complex. Therefore, by preventing downregulation of EEF1A via miR-33a, the virus ensures that its replication can occur efficiently (Chen et al., 2016). In the case of poliovirus, replication relies on the recruitment of heterogeneous nuclear ribonucleoprotein C1/C2 (hnRNP C) to the viral replication machinery. Shim et al. (2016) identified miR-555 as a miRNA with potent antiviral properties in a high-throughput overexpression screen, and showed that hnRNP C was regulated by this miRNA. However, in this case, it is not known whether the virus modulates the expression of this miRNA during infection.

As a last example, the enterovirus EV71 also partially relies on the downregulation of the cellular miRNA miR-197, which is involved in the regulation of the nuclear import factor RAN (Tang et al., 2016). RAN is important for the import of the viral protein 3D and 3DC, which plays an important role for the virus replication, although in an indirect manner.

#### Translation

Viruses also rely on the host machinery for translation of viral proteins. The *Picornaviridae* family gathers viruses with a positive sense RNA genome whose protein expression is cap-independent and rather depends on an IRES (Martínez-Salas et al., 2015). To hijack the cellular machinery and use it on their advantage, these viruses shut-off the cap-dependent translation. miRNAs can also be involved in this process. For instance, miR-141 has been the first miRNA described to participate in such a process and to have a positive effect on the viral infection (Ho et al., 2011). During EV71 infection, miR-141 expression is upregulated and the expression of the initiation factor eIF4E is repressed. Interestingly, unlike other translation initiation factors, eIF4E in necessary for cap-dependent translation but not for cap-independent translation. Ho and collaborators have shown by Chromatin ImmunoPrecipitation (ChIP) assays that the early growth response 1 (ERG1) transcription factor, which is induced upon infection, enhances miR-141 expression leading to the silencing of eIF4E, promotion of translational switch and increased viral production. This is a novel mechanism of translational switch during EV71 infection involving miR-141.

#### Immune Response

Innate immunity is the first line of defense against viral infection. After recognition of viral specific elements by different receptors an antiviral response sets in place triggering complex signaling pathways that lead to the activation of interferon response and production of cytokines. The involvement of miRNAs in the control of immune response has been studied extensively (see Taganov et al., 2007 for review), and it is not surprising that some miRNAs are specifically involved during viral infection to regulate the cellular response. This is the case of miR-144, which we mentioned earlier, and which interferes with the immune response allowing an increased replication of different RNA viruses. After validation of computational prediction, miR-144 was shown to act as a positive regulator of viral infection by targeting TRAF6 mRNA thereby regulating IRF7-mediated immune response. Furthermore, *in vivo* assays performed on mice where miR-144 expression is suppressed showed reduced viral infection suggesting a possible use of miRNAs to modulate the host immune response (Rosenberger et al., 2017). Another interesting example is miR-485, which is positively upregulated during infection by NDV or IAV and upon treatment with synthetic dsRNA. The upregulation of this miRNA results in a reduction of interferon and inflammatory cytokines such as IL6. The mechanism by which miR-485 is able to modulate antiviral response is through direct targeting of the RIG-I 3′ UTR thereby affecting the rest of the signaling cascade and the antiviral gene expression. Interestingly, upon influenza H5N1 infection at a high viral load, miR-485 action switches to control the virus and directly targets the viral gene PB1 coding for a RNA-dependent RNA polymerase protein required for replication (Ingle et al., 2015). The regulation of miRNA expression upon viral infection can also be due to the induction of the interferon pathway, which means that some interferon-regulated miRNAs can play a broad antiviral role (Pedersen et al., 2007). More recently, Robertson et al. (2016) identified miR-342 as an important regulator of multiple targets involved in the sterol pathway that is important in the macrophage interferon antiviral response. The authors also showed that this interferon-modulated miRNA was playing an antiviral role against several unrelated viruses (Robertson et al., 2016).

#### Apoptosis

The ultimate outcome of an efficient immune response is the induction of programmed cell death, which is set in place to avoid further viral production in a cell when the virus has not been successfully cleared. At the same time, apoptosis induction in late stages of infection can also help the virus to spread in the extracellular milieu or in neighboring cells. Following WNV infection, the expression of miR-6124 (referred to as Hs-154 in this paper) is induced. The authors found that this miRNA targets CTFC and ECOP, both anti-apoptotic factors, leading to apoptosis (Smith et al., 2012). Thus, in this case, cell death induction by WNV is partially mediated by the induction of a cellular miRNA. Another miRNA, miR-532, is involved in WNV replication in human cells as well as in mice brain and also interferes with apoptosis during infection. It was found to target TAB3, a protein involved in the NF-kB pathway, known to inhibit apoptosis and promote cell survival. Furthermore, it can also target SESTD1, a phospholipid binding protein localized in the plasma membrane and involved in the regulation of calcium transport by activating Ca^2+^ channels TRPC4 and TRPC5. During WNV infection, calcium can lead to cleavage of caspase 3, interfering with activation of FAK and ERK1/2 pathways, promoting cell survival and thus maintaining the optimal environment for the virus to complete its cycle (Slonchak et al., 2016). The last two examples perfectly illustrate the complexity of deciphering miRNA-mediated regulation during viral infection, since both pro- and anti-apoptotic functions occur simultaneously.

## Virus-Mediated Regulation of miRNA Activity

As mentioned earlier in the introduction, co-evolution between viruses and their host results in an equilibrium to maintain the host alive despite viral infection. It is thus logical that in cases where cellular miRNAs have an antiviral effect, viral counter measures have been observed. In addition, in some cases, the host can also benefit from a modulation of miRNA activity in response to an infection. Several strategies have been reported that are either unspecific, in the sense that the miRNA biogenesis machinery or all cellular miRNAs are impacted upon viral infection; or targeted, since one or a few miRNAs are regulated specifically.

### Non-specific Modulation of miRNA

One of the best ways to prevent detrimental targeting by small RNAs is to get rid of all of them. This seemingly radical approach is employed by the vaccinia virus (VACV), a large DNA genome virus, which expresses a poly-A polymerase known as VP55. This viral enzyme was known to be involved in the polyadenylation of the viral mRNAs until Backes et al. (2012) showed that it could also mediate poly A tailing of cellular miRNAs. The addition of these residues apparently results in the degradation of mature miRNAs and can be prevented when the RNA is 2′-*O*-methylated. The authors hypothesize that this strategy of inducing decay of small RNAs initially appeared in arthropod-infecting poxviruses, to remove siRNAs generated by Dicer to control infection. As a counter-counter-measure, insects would then have evolved to modify siRNAs by 2′-*O*-methylation in order to avoid their degradation. In mammals though, miRNAs are not 2′-*O*-methylated, and the benefit of degrading miRNAs for VACV remains elusive, although it could be indeed to prevent direct targeting of viral mRNAs by the host miRNAs. Adenovirus VA1 RNA was also reported some time ago as being able to perturb miRNA biogenesis by saturating Exportin 5 and Dicer (Lu and Cullen, 2004). This very abundant, multifunctional, viral non-coding RNA adopts a hairpin structure, which is also important for the inhibition of PKR or OAS1 (see Vachon and Conn, 2016 for review). The real importance of miRNA biogenesis alteration has not been readily addressed to date.

In other cases, a virus-induced response can lead to the alteration of key factors involved in miRNA activity, but with a net result that is negative for the virus. Thus, Argonaute proteins can be poly-ADP-ribosylated during stress conditions (Leung et al., 2011), including viral infection (Seo et al., 2013), which results in their inactivation. Although at first, it seems that limiting RISC activity might be beneficial for the virus if some miRNAs played antiviral roles, it is in fact the opposite. Indeed, Seo et al. (2013) showed that some ISGs are regulated by miRNAs of the miR-17 family, and therefore blocking Argonaute proteins by ADP-ribosylation results in the de-regulation of these antiviral factors to allow the cells to mount an effective response. Interestingly, antiviral signaling factors such as MAVS or RNase L are involved in the post-translational modification of AGO proteins, although the exact mechanism is not known. The identification of viral factors that could modulate the ADP-ribosylation machinery would be a nice validation of the importance of this pathway in the interplay between viruses and miRNAs.

### Specific Regulation of miRNAs by Viruses

As we discussed above, there are cases where it is important for the virus to keep a miRNA target sequence, when it is beneficial during its replication cycle. This is the case with HCV, which has evolved to select a binding site for miR-122, thereby allowing the recruitment of AGO2 at the 5′ extremity of the viral genome to protect it from degradation. However, when a cellular miRNA directly targets a viral RNA, it does not make sense for the virus to maintain the target sequence if it limits its fitness. This is especially true for RNA viruses, which have a greater capacity to evolve quickly. Nevertheless, when the miRNA effect is indirect, it is more complicated to affect the targeting by the miRNA to get rid of its unwanted effect. There are cases where acting indiscriminately on the miRNA biogenesis machinery, as mentioned in the previous part, is not an option, either because the virus relies on this machinery to make its own miRNAs, or because it will have a too strong impact on the longer term. The mouse cytomegalovirus (MCMV) is a betaherpesvirus, which expresses a number of miRNAs (Dölken et al., 2007), and which was also shown to be negatively impacted by overexpression of the cellular miR-27 (Buck et al., 2010). However, in normal conditions, miR-27 does not have any effect on MCMV mainly because upon infection the level of the mature miRNA is dramatically reduced. This observation suggested that the virus somehow developed a strategy to actively degrade this miRNA since the level of the miRNA primary transcript or of the pre-miRNA was unaffected (Buck et al., 2010). It was later found that the virus expressed a transcript that contains a binding site for miR-27 that acts as a decoy to titer out the miRNA and induce its degradation (Libri et al., 2012; Marcinowski et al., 2012). This phenomenon, referred to as target RNA directed miRNA decay (TDMD), is known to occur when a target is almost perfectly complementary to the miRNA (Ameres et al., 2010; de la Mata et al., 2015; Haas et al., 2016). In the case of MCMV, it was indeed confirmed that being able to degrade miR-27 is important in the time course of *in vivo* infection, but it is possible that the virus does require regulation by miR-27 in the early stages of infection. Indeed, the level of expression of the viral transcript involved in the miRNA decay is key in this process, and it is only in late stages of infection that this RNA reaches sufficient levels in order to be able to fulfill its miRNA degradation role. Other viruses, such as Herpesvirus Saimiri (HVS) and human cytomegalovirus also make use of this strategy to remove specific miRNAs (Cazalla et al., 2010; Lee et al., 2013). The case of HVS is interesting since it was later shown that the virus also makes use of one of its own non-coding RNA, known as HSUR (Herpesvirus Saimiri U RNA), not only to repress some but also to recruit other miRNAs to specific targets through dual binding to cellular mRNAs and miRNAs (Gorbea et al., 2017).

## Clinical Application of miRNAs in Viral Infection and Therapy

Using miRNAs as a tool to modify gene expression still holds many promises for the treatment of different human pathologies (Chakraborty et al., 2017) and, recently, it has become especially attractive in the field of infectious diseases. Indeed, miRNAs are very interesting molecules in the context of antiviral therapy as they show low immunogenicity and, for the ones that show cross-species conservation, they can be tested in various animal models in preclinical studies. Despite the availability of many different methods to inhibit (Li and Rana, 2014) or overexpress a given miRNA (Yang, 2015) *in vivo*, delivery remains a challenge for miRNA-based therapy in future clinical applications.

In addition, the silencing ability of the endogenous miRNA machinery is currently harnessed for the development of live attenuated vaccines. For instance, insertion of a tissue specific miRNA binding site in a given viral genome can disable viral replication in a specific cell type. Similarly, miRNA direct binding to its target sequence can be engineered to assure selectivity in viral cell tropism and to diminish toxicity in the case of oncolytic viruses.

### miRNA Targeting as an Antiviral Therapy

An exciting example of miRNA-based treatment for antiviral therapy is represented by the use of inhibitors of miR-122 in HCV infection. An initial study showed that systemic delivery of a 15-nucleotide locked nucleic acid (LNA) oligonucleotide with phosphorothioate modifications (later on named SPC3649 or Miravirsen), complementary to the 5′ end of miR-122, results in sequestration of the endogenous miRNA in non-human primates without any associated toxicity (Elmen et al., 2008). Soon after, silencing of miR-122 by the antisense oligonucleotide Miravirsen was also achieved in chimpanzees with chronic HCV infection and provided long-lasting viral suppression (Lanford et al., 2010). Janssen et al. (2013) conducted a phase 2a study in chronic HCV infected patients who received 5-weekly injections of Miravirsen. The treatment resulted in a prolonged and dose-dependent reduction in HCV RNA levels (Janssen et al., 2013; van der Ree et al., 2014). More recently, assessment of miR-122 plasma levels in chronic HCV infected patients upon Miravirsen treatment demonstrated a significant, specific and prolonged decrease in miR-122 expression, close to detection limits in some cases. However, this was not always accompanied by a substantial reduction in the viral load (van der Ree et al., 2016). Finally, a *N*-acetylgalactosamine conjugated antisense oligonucleotide for miR-122, named RG-101, was developed to increase miR-122 sequestration by improving delivery in hepatocytes. In a phase 1B trial, RG-101 treatment resulted in substantial viral load reduction in all treated patients within 4 weeks (van der Ree et al., 2017).

An efficient delivery of miRNA mimic and/or inhibitor is crucial for *in vivo* therapy. Although not yet at the stage of clinical trials, *in vivo* studies in animals on the antiviral activity of miR-181 and miR-130 against PRRSV support the potential of miRNA intranasal inhalation for future therapies against respiratory viruses. Intranasal delivery of chemically modified miR-181 mimics in pigs caused a slower progression of PRRSV infection thus conferring some temporary protection against the virus (Guo et al., 2013). Moreover, piglets subjected to miR-130 intranasal delivery were able to control the infection and survived longer than controls infected with a lethal dose of the virus (Li L. et al., 2015).

In a very recent study, Peng et al. (2018) demonstrated that the intranasal administration of a combination of five chemically modified miRNA mimics corresponding to highly expressed miRNAs in respiratory epithelial cells was able to target the viral RNA, synergistically suppressed H1N1 replication and protected mice from viral infection. Another study showed that the neurotropic virus JEV induces miR-301 expression in neuronal infected cells which in turns impairs the antiviral host response. *In vivo* inhibition of miR-301 by intracranial injection of modified miR-301a morpholino (see **Table 1**) restores the IFN response improving survival of JEV infected mice by enabling IFNβ production, thereby restricting viral propagation (Hazra et al., 2017). Although very promising for the treatment of neurotropic viral infection, crossing the blood-brain barrier represents an additional difficulty for small RNA-based approaches in the future that will have to be addressed.

### Attenuated Vaccines via miRNA-Directed Targeting

Live attenuated vaccines against human viral pathogens are amongst the most successful interventions currently available (Minor, 2015). The natural capacity of cellular miRNAs to inhibit viruses through direct targeting of viral RNAs can be exploited to generate new attenuated vaccines in a tissue specific manner by incorporating cell-specific miRNA target sequences into their genomes. Such a strategy can be very useful to design safe and effective live vaccines. Thus, as a proof-of-principle, insertion of complementary sequences for the neuronal-specific miR-124 into the poliovirus genome restricts its tissue tropism in mice and prevent pathogenicity of the attenuated viral strain (Barnes et al., 2008).

On the same line, an alternative approach based on miRNA-mediated gene silencing was applied to increase attenuation and improve vaccine safety for influenza A virus (Perez et al., 2009). The current live attenuated IAV vaccine is grown in eggs by conferring temperature sensitivity to the virus. Perez and colleagues engineered the virus by inserting non-avian miRNA responsive elements that mediated attenuation in mice, but not in eggs.

Finally, a recent study demonstrated that production of an IAV engineered to be targeted by miR-21, a ubiquitously expressed miRNA, is very efficient in a cell line knocked-out (KO) for this miRNA, while it is broadly attenuated in cells from a range of species across susceptible hosts including humans. The miR-21 KO cell has the potential to be used as a vaccine platform to build and grow viruses targeted by miR-21 and replace the common egg-based approaches for vaccine production (Waring et al., 2017).

### Restriction of Viral Tropism in Cancer Gene Therapy

Modification of viral tropism is not only interesting for the development of live miRNA-attenuated vaccines, but also to develop safer replication-competent oncolytic viruses (Kaufman et al., 2015). Oncolytic viruses preferentially replicate in cancer cells and in turn trigger the activation of immune response against the tumor. However, as a side effect, they can induce toxicity in normal tissues. To overcome this issue, target sequences complementary to a specific miRNA can be integrated into the viral genome to reduce replication in normal cells, while maintaining the oncolytic potential in tumor cells. This was reported for the oncolytic picornavirus coxsackie A21 that causes lethal myositis in tumor-bearing mice. Addition of binding sites for the muscle-specific miR-206 and miR-133a reduced myotoxicity while maintaining oncolytic properties (Kelly et al., 2008).

Another example is given by the miRNA-mediated attenuation of Semliki Forest Virus (SFV) neurovirulence in mice. Since SFV is able to infect cells of the central nervous system (CNS), it has been of particular interest in viral therapy of brain tumors but on the other hand additional measures are needed to restrict viral replication in neurons. Neuropathogenicity of Semliki Forest virus can be selectively attenuated by inserting in its genome binding sites for the neuron-specific miR-124, which makes it a promising tool for cancer therapy in the brain (Ylosmaki et al., 2013).

Finally, the altered expression of specific miRNAs representing a hallmark of tumor cells has been used as a way to achieve tumor-specific replication of engineered oncolytic viruses. By taking advantage of the global decreased expression of Let-7 in tumor cells, Edge and colleagues demonstrated that incorporation of Let-7 miRNA complementary sequences within VSV genome eliminates replication and associated toxicity in normal cells but allows growth in cancer cells both *in vitro* and *in vivo* (Edge et al., 2008).

## Discussion

In this review, we have extensively covered the various aspects involving miRNAs during viral infection. We deliberately chose to focus on cellular miRNAs in order to avoid to over-complicate our message. However, it is important to keep in mind that some viruses encode their own miRNAs (see Kincaid and Sullivan, 2012 for a recent review), which adds another layer of regulation mediated by the virus. These viral small RNAs are clearly important during infection since they have been selected by the virus to modify the cell environment in a non-immunogenic manner. But, how important are host-encoded miRNAs during viral infection? As we discussed above, there are some cases where there is no doubt that one cellular miRNA has been selected to create an evolutionary advantage for the virus, and the best illustration of this is the role played by miR-122 during HCV infection. However, the contribution of host miRNAs as negative regulators of viruses remains a debated topic. Bogerd et al. (2014) postulated that the replication of many human viruses was unaffected by endogenous miRNAs. The authors generated a Dicer knock-out cell line that was then infected with a variety of viruses such as DENV, WNV, yellow fever virus, Sindbis virus, measles virus, influenza A virus, VSV or HIV-1 and compared the level of virus production with the parental cells infected with the same viruses (Bogerd et al., 2014). Since no significant difference between cells depleted of or expressing Dicer was observed, they concluded that host miRNAs had no impact on virus replication. One limitation of the study though was the choice of one single cell type (HEK293T cells), which would not take into account tissue-specific expression of certain miRNAs. However, for future studies, it will be important to consider these observations when assessing the effect on a given virus of miRNAs known to be expressed in HEK293T cells.

Another group also used a global approach to show that depletion of miRNAs did not have strong effects in term of antiviral response. Aguado et al. (2015) expressed the vaccinia virus VP55 protein that we mentioned previously as able to induce degradation of all cellular miRNAs and they measured the effect on the cellular response to viral infection. One of their main conclusions was that in cells expressing VP55, and thus devoid of mature miRNAs, the acute response to a challenge with dsRNA or IFN-β was overall unaffected. However, one clear difference in cells without miRNAs was an increased cytokine production when challenged, which would indicate that miRNAs do play important roles during chronic infection and activation of the immune response. In this study, the authors did not assess the impact of VP55 expression on a *bona fide* viral infection and only used synthetic challenges, but the expression of VP55 might have other miRNA-independent effects on viral replication that would complicate the interpretation of the results.

Although the opposite cannot be strictly ruled out, it only seems logical that cellular miRNAs would not play critical roles in acute infections, since typically a miRNA-mediated effect is more that of a fine-tuning than of an on/off switch. In addition, even if a cellular target playing an important role in antiviral response was strongly regulated at the mRNA level, the half-life of the protein would have to be very short in order to result in a meaningful effect in the early phase of infection. Therefore, when assessing the role of cellular miRNAs, we can safely say that they are crucial when they are proviral, or when a longer, persistent infection is established. We have also seen that viruses do have an impact on miRNA expression pattern, which is in favor of a real importance of at least these specific miRNAs during infection. Finally, with the advent of techniques that allow to validate the physical interactions between miRNAs and viral genomes, a number of examples where the miRNA acts by binding directly to the pathogen RNA has been recently reported (Scheel et al., 2016). Again, these examples only make sense biologically when the effect is beneficial for the virus, otherwise the virus will find a way to prevent inhibition (Cullen, 2013).

Can we harness the results obtained on the study of miRNA/virus interactions to design novel therapeutic approaches? We described the use of antisense oligonucleotides to block proviral miRNAs, which is the most straightforward and easy to implement way toward new drugs. But, miRNAs can also be used as an entry gate into regulatory networks that could be explored to find new unconventional therapeutic targets.

## Author Contributions

All authors listed have made a substantial, direct and intellectual contribution to the work, and approved it for publication.

## Conflict of Interest Statement

The authors declare that the research was conducted in the absence of any commercial or financial relationships that could be construed as a potential conflict of interest. The handling Editor declared a shared affiliation, though no other collaboration, with the authors.

## Footnotes

1 www.mirbase.org
